# Supplementary figures and images for: A polarized cell system amenable to subcellular resolution imaging of influenza virus infection
Source: PLoS One. 2024 Jan 25;19(1):e0292977. doi: 10.1371/journal.pone.0292977 (PMC10810476; doi:10.1371/journal.pone.0292977)

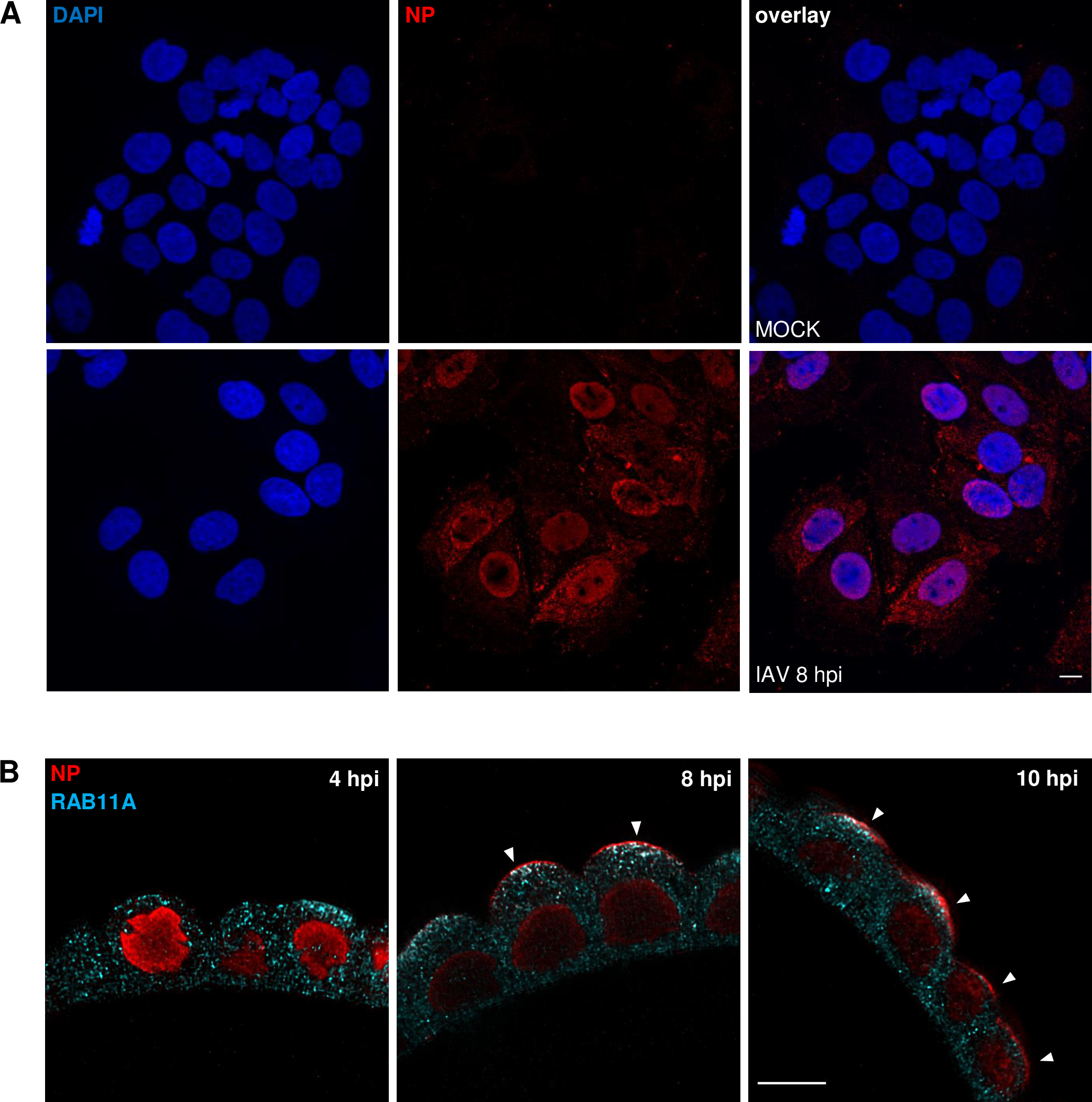

Supplement: S1 Fig — (TIF) [file pone.0292977.s001.tif]

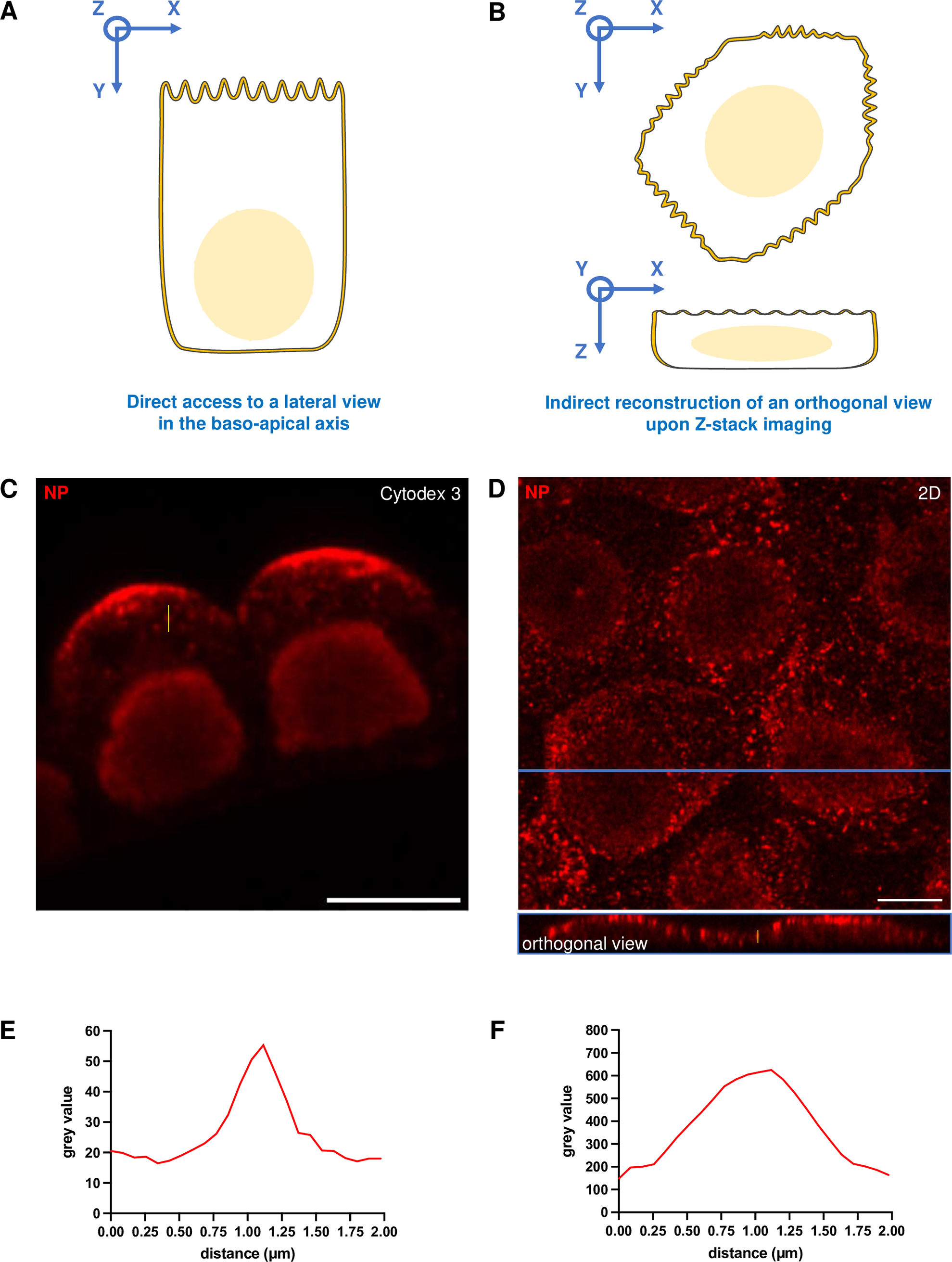

Supplement: S2 Fig — (TIF) [file pone.0292977.s002.tif]
