# Supplementary material for: A polarized cell system amenable to subcellular resolution imaging of influenza virus infection
Source: PLoS One. 2024 Jan 25;19(1):e0292977. doi: 10.1371/journal.pone.0292977 (PMC10810476; doi:10.1371/journal.pone.0292977)
Supplement: S1 Protocols — (PDF) [file pone.0292977.s003.pdf]

STEP-BY-STEP PROTOCOL

A polarized cell system amenable to subcellular resolution imaging of influenza virus infection

Jean-Baptiste Brault[1*], Catherine Thouvenot[2], Magda Cannata Serio[1#], Sylvain Paisant[1], Julien Fernandes[3], David Gény[4], Lydia Danglot[4,5], Adeline Mallet[2], Nadia Naffakh[1*]

[1] Institut Pasteur, Université Paris Cité, CNRS UMR 3569, RNA Biology of Influenza Viruses, Paris, France

[2] Institut Pasteur, Université Paris Cité, C2RT, Ultrastructural BioImaging Unit, Paris, France

[3] Institut Pasteur, Université Paris Cité, C2RT, Unit of Technology and Services Photonic BioImaging, Paris, France

[4] Institute of Psychiatry and Neuroscience of Paris (IPNP), INSERM U1266, NeurImag facility, Université Paris Cité, Paris, France

[5] Institute of Psychiatry and Neuroscience of Paris (IPNP), INSERM U1266, Membrane Traffic in Healthy and Diseased Brain team, Université Paris Cité, Paris, France

# current address: Institut Curie, PSL Research University, CNRS UMR144, Paris, France

* corresponding authors : jean-baptiste.brault@pasteur.fr, nadia.naffakh@pasteur.fr

SEEDING AND POLARIZATION OF CACO-2/TC7 CELLS ON CYTODEX 3 BEADS

1. Prepare the repellent layer and seed the Caco-2/TC7 cells

   1.1 Dissolve the Gelrite solution (0.8% Gelrite + 0.1% $MgSO_4$ x7H_2O w/v in PBS) in the microwave oven

   1.2 Distribute the Gelrite solution (2.5mL/well) in a 35 mm dish or a well of a 6-well plate. Let it solidify and cool down at room temperature for about 10 minutes.

   1.3 Wash a 100 mm diameter culture dish of subconfluent Caco-2/TC7 cells with 5 mL of PBS

   1.4 Add 1mL of trypsin and incubate 5 mn at 37°C

   1.5 Add 9 mL of DMEM supplemented with 10% fetal calf serum, 100 U/mL penicillin and 100 µg/mL streptomycin (D10 medium). Pipet up and down to homogenize the cell suspension

   1.6 Count the cells. Prepare 2 mL of a cell suspension at 2.25 x 10^5 cells/mL

   1.7 Add 50µL of the Cytodex 3 suspension (1 g/100 mL~1500 beads/50 µL) to the cell suspension, mix gently and add to the Gelrite layer. Place into the cell incubator onto a rubber pad to attenuate the incubator's vibrations.

2 The next day, homogenize the beads suspension by gently pipetting up and down 5x with a 1000 µL pipetman tip, place them back in the cell incubator

3 After three days, filter out the non-attached cells

   3.1 Place a 70µm cell strainer over a 50mL tube

   3.2 Collect the beads suspension and pass it through the cell strainer. Discard the flow-through

   3.3 Flip the filter with sterile tweezers over the Gelrite layer, add 2mL of fresh D10 medium on the top of the flipped filter to recover the beads

   3.4 Change the medium twice a week by removing the medium carefully while leaving the beads at the surface of the Gelrite layer, and replacing it with fresh D10 medium. Keep going for 10-15 days.

INFLUENZA A VIRUS INFECTION (in a BSL2+ laboratory)

4. Transfer the Cytodex beads from the Gelrite layer to a 15mL tube. Let them sediment at the bottom of the tube.

5. Remove gently the medium, add 1mL of prewarmed (37°C) DMEM for washing, and let the beads sediment at the bottom of the tube

6. Repeat the washing step once, then transfer the beads to a 1.5mL tube.

7. Let the beads sediment and remove gently the medium, resuspend the beads in 250µL of a suspension of Influenza A virus (A/WSN/1933(H1N1)) at a high titer. Given the estimated numbers of ~ 1500 beads/sample and ~400 cells/bead, a ~2,5 x10^7 PFU/mL titre will lead to an MOI of ~10 PFU/cell.

8. Incubate the beads at 37°C for 1 hr, and gently tap the tube with fingertip every 15 mn to resuspend the beads.

9. Let the beads sediment at the bottom of the tube, remove the viral inoculum, add 1mL of PBS for washing and let the beads sediment at the bottom of the tube.

10. Remove the PBS, add 1 mL of DMEM supplemented with 2% FBS, resuspend the beads and place them in a well of a 12-well culture plate. Place the plate in a cell incubator at 37°C for the selected period of time (typically, 2-6 hours to investigate early stages of the viral life cycle, 8-24 hours to investigate late stages).

CONFOCAL IMAGING

11. Fix the cells

- 11.1 Remove the medium carefully while leaving the beads at the bottom of the well. Resuspend the beads in 250µL of PBS-4% PFA. Incubate at room temperature for 20 mn..
- 11.2 Transfer the beads suspension in a 1.5mL tube. Let the beads sediment at the bottom of the tube. Remove gently the PBS-4% PFA, add 1mL of PBS for washing, and let the beads sediment at the bottom of the tube.
- 11.3 Repeat the washing step twice, then transfer the beads to a 1.5mL tube. At this stage, the sample can be considered no-longer infectious and can therefore be manipulated outside the BSL2+ facility.
- 11.4 Let the beads sediment at the bottom of the tube. Remove the PBS and add 1mL of PBS-50mM NH_4Cl for 10 minutes at room temperature, to quench the residual PFA.
- 11.5 Wash the beads 2x with 1mL PBS as described above

12. Immunostain the cells

- 12.1 Let the beads sediment at the bottom of the tube. Remove the PBS and add 500µL of the blocking buffer. Place the tube on a spinning wheel at 4°C for 1h, with a rotation speed of 15 rpm.
- 12.2 Let the beads sediment at the bottom of the tube. Remove the blocking buffer and add 250 µL of the primary antibody diluted in the immuno-staining buffer. Place the tube on a spinning wheel at 4°C overnight, with a rotation speed of 15 rpm.
- 12.3 The next day, wash the beads 3x with 1mL of PBS-0.05% Tween 20 as described above

12.4 Let the beads sediment at the bottom of the tube. Remove the PBS-0.05% Tween 20 and add 250µL of the secondary antibody diluted in the immunostaining buffer*. Place the tube on a spinning wheel at 4°C for 2 h, with a rotation speed of 15 rpm.

12.5 Wash the beads 2x with 1mL of PBS- 0.05% Tween 20 as described above

12.6 Wash the beads 2x with 1mL of PBS as described above

12.7 Wash the beads 2x with 1mL of distilled water as described above

12.8 Let the beads sediment at the bottom of the tube and remove the water. Use a very thin 10µL pipetman tip to remove residual water and to dry out the beads pellet. Immediately add 50µL of Vectashield mounting medium over the beads pellet, avoiding the formation of bubbles.

12.9 Gently tap the tube with fingertip to resuspend the beads.

12.10 Place an adhesive secure-seal spacer on a clean glass microscope slide.

12.11 Cut the extremity of 200µL pipetman tip to widen it, gently pipet out the beads resuspended in Vectashield mounting medium, avoiding the formation of bubbles. Drop them at the center of the spacer on the glass microscope slide.

12.12 Cover the drop with a sterile #1.5 round glass microscope coverslip. Protect from light and let at room temperature overnight and seal with nail polish

13a. Acquire confocal images.

13.1 Select the 40x NA 1,3 oil immersion objective and apply adapted oil.

13.2 Install sample on the slide holder of the microscope.

13.3 Create the lightpath configuration corresponding to the staining used. Create three imaging sequence:
1) DAPI : Excitation 405 nm, Emission : 416-501 nm
2) PALS-1 : Excitation 598 nm, Emission : 608-643 nm
3) NP : Excitation 646 nm, Emission : 656-705 nm

13.4 Find your sample, adjust focus.

13.5 Laser power and gain settings will be set according to the intensity of the labelling in order not to saturate the signal.

13.6 Define your Z-stack by selecting the top and the bottom of your sample. Define Z step size to 0,35 µm

13.7 Set acquisition parameters: Set frame size to 1024x1024 pixels (0,3 µm/pixel), scan speed to 400 Hz in a bi-directional mode.

13.8 Start image acquisition.

14a. Acquire confocal images at high magnification.

14.1 Select the HC PL APO 93x NA 1,3 glycerol immersion objective and apply adapted glycerol.

14.2 Install sample on the slide holder of the microscope.

14.3 Adapt the motorized objective colar to match the sample index, using reflection mode in XZY mode.

14.4 Create the lightpath configuration corresponding to the staining used. Create three imaging sequence:
1) Atto 647N : Excitation 670 nm, Emission : 654-719 nm
2) AlexaFluor 594 : Excitation 594 nm, Emission : 604-641 nm
3) Stargreen : Excitation 470 nm, Emission : 505-561 nm

14.5 Find your sample, adjust focus.

14.6 Laser power and gain settings will be set according to the intensity of the labelling in order not to saturate the signal. HyD detectors are used to favor sensitivity.

14.7 Define your Z-stack by selecting the top and the bottom of your sample. Define Z step size to 0,18 µm

14.8 Set acquisition parameters: scan speed to 400 Hz in a bi-directional mode, Frame Average2. Adapt zoom, matrix size and Z step to be in ideal sampling (here pixel size 85 nm).

ELECTRON MICROSCOPY IMAGING

15. Fix the cells.

15.1 Remove the medium carefully while leaving the beads at the bottom of the well. Resuspend the beads in 250 µL of PBS-3% PFA-0.1% glutaraldehyde. Leave at 4°C overnight.

15.2 Transfer the beads suspension in a 1.5mL tube. Let the beads sediment at the bottom of the tube. Remove gently the PBS-3% PFA-0.1% glutaraldehyde, add 250µL of PHEM-2.5% glutaraldehyde. Leave at 4°C for 24 h. At this stage, the sample can be considered no-longer infectious and can therefore be manipulated outside the BSL2+ facility.

15.3 Let the beads sediment at the bottom of the tube. Remove gently the PHEM-2.5% glutaraldehyde, add 1mL of PHEM, incubate for 5 mn at room temperature.

15.4 Repeat the PHEM washing 2x.

16. Perform uranyl acetate staining.

16.1 Let the beads sediment at the bottom of the tube. Remove gently the PHEM, add 250µL of the PHEM-1% Osmium tetroxide-1.5% potassium ferrocyanide post-fixation buffer. Incubate for 1h at room temperature.

16.2 Wash 3x in PHEM as described above.

16.3 Wash 3x in filtered distilled water, with 5 mn incubation times as described above

16.4 Let the beads sediment at the bottom of the tube. Remove gently the water, add 250 µL of water supplemented with 0.2% tannic acid. Incubate 30 mn at room temperature.

16.5 Wash 3x in filtered distilled water, with 5 mn incubation times as described above.

16.6 Let the beads sediment at the bottom of the tube. Remove gently the water, add 250 µL of water supplemented with 2% osmium. Incubate 30 mn at room temperature.

16.7 Wash 3x in filtered distilled water, with 5 mn incubation times as described above.

16.8 Let the beads sediment at the bottom of the tube. Remove gently the water, add 250 µL of 25% ethanol in water (v/v)- 1% Uranyl Acetate. Incubate 1 h at room temperature (protect from light).

17. Embed the samples.

17.1 Let the beads sediment at the bottom of the tube. Remove gently the uranyl acetate solution. Add 1 mL of 50% Ethanol in water (v/v). Incubate 5 mn at room temperature

17.2 Repeat step 12.9 with 75% ethanol in water (v/v).

17.3 Repeat step 12.9 with 100 % ethanol.

17.4 Prepare the Epoxy resin at room temperature (https://www.emsdiasum.com/docs/technical/datasheet/14120).

17.5 Let the beads sediment at the bottom of the tube and remove gently the ethanol with a micropipette.

17.6 Add 200 µL of Epoxy resin onto the beads and homogenize slowly with back-and-forth movements with the micropipette.

17.7 Let the beads sediment at the bottom of the tube. Remove 150 µL of Epoxy resin and add 150 µL of fresh Epoxy resin.

17.8 Wait 3 hours of impregnation time with the tubes open under the chemical hood.

17.9 Incubate overnight at room temperature to allow polymerization of the resin.

18. Section the samples with an ultramicrotome.

18.1 Use a fresh blade to trim away excess resin from the block underneath the embedded compound eye under the stereomicroscope. Shape the upper part into an equilateral trapezoid, and shape the lower part into a square base.

18.2. Place the shaped block into the chuck on the ultramicrotome and tighten it firmly.

18.3. Place the HISTO diamond sectioning knife on the sectioning knife holder stage on the ultramicrotome

18.4. Add a little excess water to the groove of the diamond sectioning knife to make it convex and stop when the full length of the diamond blade is soaked.

18.5. Gently draw a portion of the water under the microscope with a syringe or Pasteur pipette. Adjust the lightning system so that when the liquid surface becomes concave, a curved surface reflecting light can be seen, which is the correct liquid surface and appears white under the microscope. As the liquid evaporates during sectioning, distilled water should be added to ensure the correct liquid level.

18.6. Adjust the knife angle so that the two parallel sides of the trapezoid sample are parallel to the blade.

18.7. Move the knife to align the available part with the block surface.

18.8. Raise the block until it stops slightly above the knife blade.

18.9. Set parameters in the control panel: feed of 1000 nm, speed of 1 mm/s, thickness 1 µm. Start the automated movement of the ultramicrotome specimen arm.

18.10. Cut semi-thin sections (1µm thick). The sections float on the water in the bowl of the HISTO diamond knife.

18.11. Collect the sections in a drop of water with a handle and place them on a glass slide.

18.12. Stain the semi-thin sections by adding a drop of 50 µL of toluidine blue (5% p/v) on the sections and incubating the samples 2 mn on a hot plate (60°C).

18.13. Look at the stained semi-thin sections on the glass slide with a photonic microscope. Repeat the steps 18.4 to 18.9 until an area of interest with beads is observed.

18.14. Halt sectioning and replace the HISTO diamond knife with an Ultra 45° diamond knife. Fill the knife reservoir with fresh distilled water. Adjust the liquid level and sectioning parameters: feed of 70 nm, speed of 1 mm/s.

18.15. Cut ultrathin sections (70 nm thick). The sections float on the water in the bowl of the Ultra 45° diamond knife.

18.16. Carefully collect 2 sections on a carboned grids.

18. 17. Place the grids with thin sections into the transmission electron microscope sample box.

19. Staining of ultrathin sections.

19.1 Prepare a parafilm of 15 cm in width and place it on a delimited radioactive area, with the clean surface of the parafilm on the upper side.

19.2. For each grid, add one drop (about 100 µL) of water supplemented with 4% Uranyl Acetate, filtered (0.22 µm), on the parafilm.

19.3. Place gently the grid (section side) on the droplet surface and let it stand for 40 min. Protect it from light.

19.4. For each grid, add three drops (about 200 µL) of distilled water on a clean area of the parafilm. Place the grid on the first of the three droplets before moving it on the second and then on the third droplet. Wait 10 s before transferring a grid to the next droplet.

19.5. For each grid, add one drop (about 100 µL) of water supplemented with 1% lead citrate, filtered (0.22 µm), on a clean area of the parafilm.

19.6. Place the grid on the top of the droplet and let it stand for 5 min.

19.7. For each grid, add five drops of fresh distilled water on a clean area of the parafilm. Wash the grids by placing them successively on the five droplets, as in step 19.4. Wait 20 s before transferring a grid to the next droplet.

19.8 After drying the grids on a filter paper, place them in the transmission electron microscope sample box.

20. Electron microscopy imaging.

20.1. Acquire images with a transmission electron microscope at 120 kV.

20.2. Collect images of cells on beads.

MATERIALS

Plastic consumables

1 mL micropipettes	Eppendorf	861172
1.5 mL Eppendorf tube	Eppendorf	0030125150
100mm diameter culture dish	Corning	353003
15 mL tube	Falcon	352097
6-well plate	Falcon	353047
12-well plate	Falcon	353043
24-well plate	Falcon	353046
Rubber pad	Applied Biosystems	N8010550

Cell culture reagents

PBS (CaCl2-)	Gibco	14190
DMEM 1X (+) 4.5g/L D-Glucose L-Glutamine (-) pyruvate	Gibco	41965
Fetal Bovine Serum (FBS) qualified	Gibco	10437
Penicillin streptomycin	Gibco	15140
Trypsin-EDTA 1X 0.05%	Gibco	25300
Gelrite	Sigma	G1910
Cytodex 3 microcarrier beads	Sigma	C3275

Cell culture medium

D10 medium: DMEM medium supplemented 10% FBS, 100 U/mL penicillin, 100µg/mL streptomycin

Reagents for electron microscopy

Paraformaldehyde solution	Euromedex	15714
Glutaraldehyde solution Grade I	Sigma	G5882-10
Osmium tetroxide	Euromedex	3000154760
Uranyl acetate	Electron Microscopy Science	22400
Ethanol absolute	Fisher scientific	10680993
Epoxy resin	LFG	14901
Lead citrate	Delta microscopies	11300
HEPES	ThermoFisher Scientific	15630080
DMP30	LFG	13600
Tannic acid	Sigma	1401-55-4
Potassium ferricyanide	Sigma	14459-95-1
EGTA	Fisher Scientific	11514736
PIPES	Sigma	P6757
PBS	Fisher scientific	12559069
Toluidine blue	Fluka AG	89640
PHEM Buffer	Delta microscopies	GT140174

Epoxy resin solution for electron microscopy:

Mix Epon (166 mL), DDSA (100mL) and NMA (84 mL) slowly for 20 min, avoid bubbles.
Add 170µl of DMP30 to 10mL of the Epoxy resin solution extemporaneously before embedding

Equipment for electron microscopy

Fine forceps Dumont, Style 7	EMS	72801-D
Electronic balance	Mettler toledo	ME2002
Razor blade (single Edge Carbon Steel)	EMS	71960
Incubator	Memmert	N/A
ultramicrotome	Leica	UC7
Formvar carboned grids 200 Mesh	EMS	FCF200-Cu-50

Diamon knife Histo 45° 8mm	LFG - Diatome	N/A
Diamon knife Ultra 45° 3mm	LFG - Diatome	N/A
Glass slide	Fisher scientific	10090431
Transmission electron microscope	ThermoFisher	Technai Biotwin T12
Stereomicroscope	Leica	N/A
Hotplate	Sartorius	N/A
Ashless quantitative filter paper (55mm)	Whatman	140055

Reagents for confocal microscopy

Paraformaldehyde 32% w/v	Thermo scientific	47377
NH_4Cl	Sigma	213330
Tween 20	Sigma	P1379
Triton X100	Sigma	T9284
Antifade mounting medium	Vectashield	H-1000
DAPI	ThermoScientific	62248
Normal donkey serum	Merck	S30
Normal goat serum	Merck	S26
Gelatin from cold water fish skin	Sigma	G7765

Buffers for confocal microscopy:

Blocking solution: 5% donkey serum, 3% goat serum, 0.25% fish skin gelatin, 0.2% Triton X100
Staining solution: 5% donkey serum, 3% goat serum, 0.125% fish skin gelatin, 0.2% Triton X100

Primary Antibodies for confocal microscopy

Mouse monoclonal anti Influenza A NP clone AA5H	BIO-RAD	MCA400 (1:500)
Rabbit polyclonal anti MPP5/PALS-1	Proteintech	17710-1-AP (1:500)
Mouse monoclonal anti ZO-1, clone 1A12	Invitrogen	33-9100 (1:100)
Rabbit polyclonal anti RAB11A	Invitrogen	71-5300 (1:100)

Secondary Antibodies for confocal microscopy

Goat anti mouse IgG Alexa Fluor555	Invitrogen	A-21424
Donkey anti rabbit IgG Alexa Fluor488	Invitrogen	A-21206
Donkey anti-rabbit IgG Alexa Fluor594	Invitrogen	A-21207
Goat anti-mouse IgG Atto 647N	Merck	50185

Equipment for confocal microscopy

Cell strainer 70µm Nylon	Corning	431751
Secure-seal spacer one well	Invitrogen	S24735
Slides Superfrost Plus Adhesion	Epredia	J1800AMNZ
Coverslips	Epredia	CB00130RAC20MNZ0
Wheel	Fisherbrand	88861050
TCS SP8 scanning confocal microscope	Leica	N/A

Softwares and algorithms

TIA	ThermoFisher	N/A
Image J	NIH	N/A
LasX	Leica	N/A
Imaris	Oxford Instruments	N/A
